# High-Dimensional Probabilistic Fingerprinting in Wireless Sensor Networks Based on a Multivariate Gaussian Mixture Model

**DOI:** 10.3390/s18082602

**Published:** 2018-08-08

**Authors:** Yan Li, Simon Williams, Bill Moran, Allison Kealy, Guenther Retscher

**Affiliations:** 1 Department of Electrical and Electronic Engineering, The University of Melbourne, Parkville, VIC 3010, Australia; simon.williams@unimelb.edu.au (S.W.); wmoran@unimelb.edu.au (B.M.); 2 Department of Geospatial Science, RMIT University, Melbourne, VIC 3000, Australia; allison.kealy@rmit.edu.au; 3 Department of Geodesy and Geoinformation, TU Wien-Vienna University of Technology, Gusshausstrasse 27-29, E120/5, 1040 Vienna, Austria; Guenther.Retscher@geo.tuwien.ac.at

**Keywords:** Multivariate Gaussian Mixture Model (MVGMM), multivariate linear regression, Expectation-Maximisation imputation, Wi-Fi localisation, Hidden Markov Model (HMM)

## Abstract

The extensive deployment of wireless infrastructure provides a low-cost way to track mobile users in indoor environment. This paper demonstrates a prototype model of an accurate and reliable room location awareness system in a real public environment in which three typical problems arise. Firstly, a massive number of access points (APs) can be sensed leading to a high-dimensional classification problem. Secondly, heterogeneous devices record different received signal strength (RSS) levels because of the variations in chip-set and antenna attenuation. Thirdly, APs are not necessarily visible in every scanning cycle leading to missing data issue. This paper presents a probabilistic Wi-Fi fingerprinting method in a hidden Markov model (HMM) framework for mobile user tracking. To account for spatial correlation of the signal strengths from multiple APs, a Multivariate Gaussian Mixture Model (MVGMM) was fitted to model the probability distribution of RSS measurements in each cell. Furthermore, the *unseen* property of invisible AP was investigated in this research, and demonstrated the efficiency as a beneficial information to differentiate between cells. The proposed system is able to achieve comparable localisation performance. Filed test results achieve a reliable 97% localisation room level accuracy of multiple mobile users in a real university campus Wi-Fi network.

## 1. Introduction

The Global Positioning System (GPS) has been widely used to provide location information in outdoor environments, but it fails to provide reliable positioning indoors [1]. Wi-Fi based localisation system has attracted considerable attention because of the prevalent deployment of Wireless Local Area Network (WLAN) infrastructure and the extensive availability of Wi-Fi enabled mobile devices, which provides a potentially low-cost way to track a mobile user in a building [2]. The vast majority of current indoor localisation systems are designed for sub-metre accuracy in position estimation, which is unnecessary for most indoor navigation applications [3]. Room-level or region-level granularity of location is sufficient for most location aware services [4,5,6,7].

Received signal strength (RSS) based Wi-Fi fingerprinting is a typical method frequently used for location estimation, since it does not need any prior knowledge of access points (APs) deployment. The idea of the fingerprint technology is to use online RSS measurements to match the fingerprint database previously generated at every location in the offline training phase. In the probabilistic fingerprint approach, a model for the statistical distribution of the signal strength for each different location is built, based on sample data collected during the training phase. In the online phase, Bayesian inference is used to calculate the probability that a user is at a certain location given a specified observation, and estimate the most likely location of the mobile device. The accuracy of the statistical distribution model directly affects the final performance of the probabilistic fingerprint positioning [8].

One major disadvantage of the fingerprint based localisation system is the intensive labour consumption in the offline survey phase. Crowdsourcing provides an efficient way to reduce the burden of training data collection by splitting the task to multiple participants. The fingerprint database is obtained by fusing the training data collected by participating devices. However, different devices have different sensor specifications and varying readings. This leads to the fingerprint annotation problem in cross-device fingerprint database that the fingerprint contributed by different devices is not compatible with each other. To support different participated devices, a linear regression calibration model is implemented to mitigate the RSS variance problem caused by the device heterogeneity.

Most existing systems assume independence between the RSS measurements at a certain position from various APs. Arising from this assumption, the observation likelihood function is calculated as the marginal conditional probability of each AP. We have demonstrated in this article that the correlation between the RSS measurements when fusing fingerprints from multiple reference points (RPs) within a cell is too high to be ignored.

Our previous research [9] employed a joint histogram model to generate the fingerprint probability distribution. In a complex and noisy open space environment, for example a university campus, a large number of APs can be scanned during both the survey and positioning phase. Matching a quantised histogram from 50 APs exactly almost never happens, thus an AP selection rule is required to get reduced-dimensional quantised states for each cell [10]. The joint histogram probability method can achieve as high as 95% room level accuracy in a real university campus based on the data collected in static mode. The problem arises for the dynamical data that the number of visible APs is typically smaller in kinematic collection mode than the set of static data. This has been demonstrated experimentally in [11], and arises from the fact that the collection of more measurements when stationary than when in motion. Other concerns are that the RSS from wireless APs are highly variable, and not all locations record signals from every AP. In addition, the set of APs operating during the training phase may not be the same as at runtime [12]. Thus, the AP selection rule will pick the AP set which is not able to represent the characteristic of the online measurements and give biased estimation of the user position. This paper builds up a high-dimensional probabilistic radio map by considering all available visible APs sensed in the training data regardless of the signal quality. This is to ensure the inclusion of every possible AP that would be see in the runtime measurement.

Most existing fingerprint-based algorithms ignore the issue of changing AP visibility at training process and runtime. A conventional method of dealing with missing data is to set a low RSS value −110 dBm [13] or assign a penalty in the matching process [14]. While the incompleteness in the sensing data can lead to bias in the estimation of parameters, we have tried a number of approaches to overcome this problem. The most successful, reported here, invokes the expectation-maximisation (EM) imputation strategy. This method, widely used in statistics, provides a method to impute the missing data and simultaneously learn the parameters from the incomplete data [15]. The key idea of EM imputation is to iteratively fill in the missing data under the current estimation of the unknown parameters and reestimate the parameters from the observed and filled-in data [16].

This paper proposes a statistical approach of localising a mobile user with room-level accuracy in a university campus wireless network. By segmenting the indoor area into several cells, the system fuses crowdsourcing RSS measurements from all visible APs collected within each cell. The system has adopted the dependency of RSS between pairs of APs caused by the change of spatial collect locations within a cell. Different devices generally provide different intensity readings due to many factors such as antenna gain and transmission power. Multivariate linear regression is used to address the RSS variance problem in the crowdsourced training data caused by the device heterogeneity. In terms of the dimension mismatch caused by variable visibility of APs, instead of discarding weak APs to reduce dimension or replace unobserved AP with a minimal constant RSS value, the EM imputation strategy is exploited to replace the missing RSS in the training data. Then, a high-dimensional probabilistic fingerprint is constructed for each cell based on the multivariate Gaussian mixture model (MVGMM) considering the correlations between APs. The Hidden Markov Model (HMM) is applied to track the mobile user, where the hidden states comprise the possible room locations and the Wi-Fi RSS measurements are taken as observations. In the positioning phase, revealing the trajectory of the user can be carried out with the Viterbi Algorithm. Besides, different rooms have both different visible and invisible AP sets, which is also a signature that can be used to differentiate between cells. By taking advantages of the “*unseen properties*” of invisible APs, a conditional probabilistic observation model is utilised to describe the likelihood of receiving a particular invisible AP set at a certain cell. The hypothesis is if an AP is invisible in the fingerprint of a cell, an online observation contains signal strength from that AP has low probability belongs to that cell. The information of invisibility of APs enabling the introduction of rigorously motivated trustworthiness for updating the conditional likelihood observation function.

The remainder of the paper is organised as follows: Section 2 briefly introduce the background and related work. Section 3 depicts the proposed system architecture. Section 4 presents the experimental results to verify the validity of the proposed algorithm. Section 5 draws the conclusion.

## 2. Related Work

The vast majority of current indoor localisation systems are designed for sub-metre accuracy in position estimation which is unnecessary for most indoor navigation [3]. Room-level or region-level granularity of location is sufficient for most location aware services [4,5,6,7].

The traditional Wi-Fi fingerprinting method involves a site survey before the test, which needs to grid the area and construct a radio map associating each location. Conventional fingerprint localisation algorithms normally average the Wi-Fi RSS measurements for each AP in stored signatures. In practice, this is not consistent with RSS fluctuations, due to the multipath effects in complex indoor environments [17]. To get real-time correction of RSS variations and fluctuations, the Differential Wi-Fi (DWi-Fi) scheme is proposed by analogy to Differential GPS (DGPS) where reference station network measurements are employed [18]. The recorded RSS measurements at user’s end are corrected and the fingerprinting database is continuously updated to encounter for the possible changes in the dynamics of the environment.

In the probabilistic fingerprint techniques, a fingerprint is the probability distribution of the signal strength given the location instead of the mean during the training phase. Some approaches assume a Gaussian distribution of signal strength [19,20,21], which is not always true as the RSS distribution tends to be left-skewed, as analysed in [22,23,24]. The Horus system infers the target location with the maximum posterior probability assuming a standard Gaussian distribution [21]. Another efficient approach to estimate the probability density distribution (PDF) is to use kernel functions [10]. Mirowski et al. extends this work by comparing the similarities between two PDFs using Kullback–Leibler divergence (KLD), and then performs localisation through kernel regression [25].

Histogram-based probabilistic methods do not assume any known distribution and is closely related to discretisation of continuous values to discrete ones [26,27,28]. However, histogram-based performance is primarily dependent on the choice of bin number and bin width. In addition, Zhang et al. pointed out that histogram-based approaches are only appropriate for low-dimensional datasets because the calculations in histogram-based techniques are exponential in the dimension of the dataset [29]. Therefore, this type of approach has low scalability to problems with larger numbers of data points and higher-dimensional spaces.

Using a subset of available APs enables reducing the number of variables and allows reliable low-dimensional quantised states for each room, which normally involves a sanity assessment to select a subset of APs for positioning [30]. The concept of “*important AP*” is proposed to select significant APs for each location where the AP with the highest RSS is denoted as the important AP [31]. This method works properly for the static data, while the problem arises for the dynamical data. As demonstrated in [11,12], the visible AP set in kinematic collection mode is typically smaller compared with the AP set in static data. Thus, the AP selection rule will pick the AP set which is not suitable to represent the characteristic of the runtime measurements and give biased estimation of the user position. This paper builds up a high-dimensional probabilistic radio map by considering all available visible APs in the training data regardless of the signal quality. This is to ensure the inclusion of every possible AP that would be sensed in the observation data instead of assigning a constant probability.

Where there are missing RSS values from some APs at some locations, a heuristic method for handling the missing data is to set a constant minimal possible RSS value [32]. In this paper, we use the EM imputation method to replace the missing values in the incomplete data. In addition, we have observed that the missing APs also provide extra information because of their *unseen* properties. A conditional likelihood observation function is presented by taking advantages of the invisibility of APs, referring to the likelihood of observing a particular invisible AP set. Similar work can be found in work [33], where an AP pickup probability is modelled using maximum entropy Gibbs distributions, indicating the beacon-visibility in each location. Bisman and Veloso neglected any unobserved or extra APs when applying the Gaussian kernel to compare different signal strengths [34]. Penalties are applied for the missing APs, as proposed in [35]. The concept of penalties is also used by the Redpin algorithm [36] where an extra bonus weight is added for common APs and an extra penalty for non-common APs.

Luo [37] suggested that the standard Gaussian distribution did not fully describe the signal strength in the indoor environment, and a more suitable fit in the probability distribution model of signal strength is based on the Gaussian Mixture Model (GMM), which infers an approximate probability distribution by a weighted mixture of Gaussian densities [38]. The WiGEM system employed the GMM to learn the signal propagation parameters for each AP [39]. The GMM is applied to model the probability distribution of the signal strength for each AP, assuming that the APs are independent at a particular position [40]. GMM is used to identify the RSS components of multipath decline separated from the line-of-sight (LOS) component in [41]. Similar work can be found in [42] where a two-node GMM is used to detect and exclude the outliers, one node for the direct path and the other one for the outliers. However, none of the systems mentioned above consider the interdependencies among the RSS measurements from the various APs. Thus, the proposed system utilises a multivariate GMM by capturing the correlation between RSS measurements from pairs of APs.

## 3. System Overview

**Figure 1:**
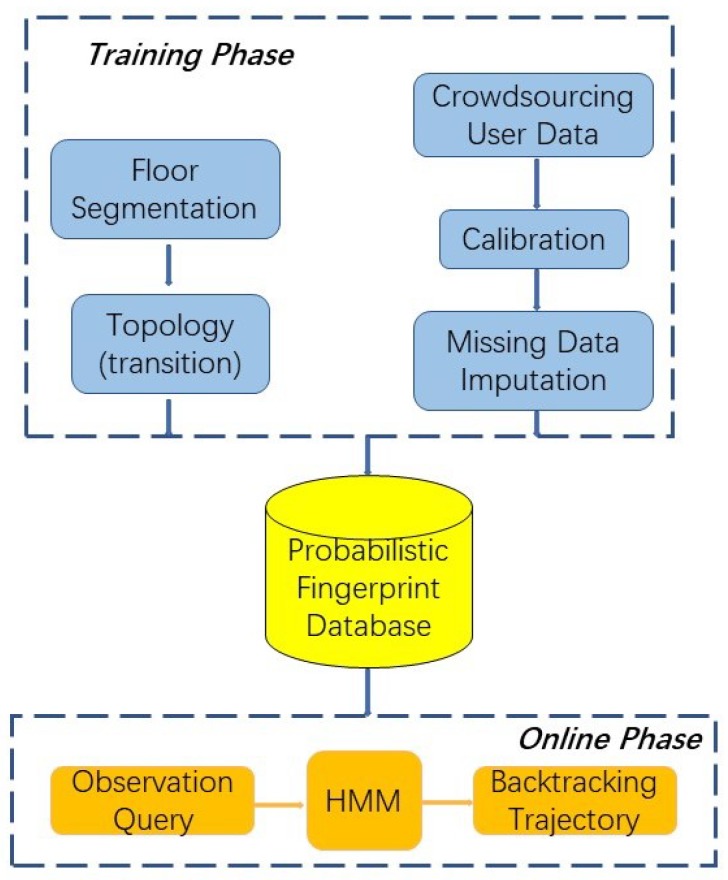
Framework of the system.

The proposed system is implemented to achieve high room level localisation accuracy and reliability. To this end, six steps need to be taken to identify the trajectory of the mobile user, as presented in Figure 1. The first step is to segment the indoor area into several cells and randomly assign multiple RPs within each cell. Then, training data collection is carried out by fusing RSS measurements taken at all RPs within each cell by all contributed devices. A multivariate linear regression is conducted to calibrate the RSS measurements collected from different devices. The missing RSS values are replaced by the new data estimated by the EM imputation method. The fifth step is to exploit the MVGMM to construct the probabilistic radio map for each cell based on the calibrated training data. Lastly, an online matching process is performed which is to fit the runtime observation into the distribution model of each cell, and feed into the Viterbi Algorithm to backtrack the trajectory of the user.

### 3.1. Building Topology

**Figure 2:**
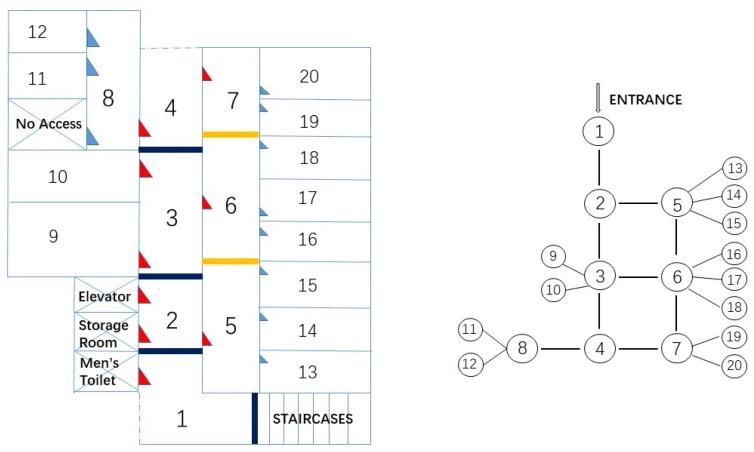
A schematic of the Bolz Hall, Ohio State University (not to scale) and topology.

Room level localisation is defined in terms of cell-based localisation, i.e., locations are represented as cells. A cell may correspond to a room, or a section of a hallway. In the test area depicted in Figure 2, for instance, the main corridor is divided into four cells. In addition, the segmentation rule classifies the floor area into three categories: rooms, corridors and entrance/exits. It constructs logical links between rooms and corridors and models the constraints to movement imposed by the building’s layout. Note that surveying the scales or true dimensions of the floor is not needed for space segmentation.

The segmentation will define the transition matrix in the HMM such that only adjacent cells have non-zero transition probability while the transition probability between isolated cells is zero. The system does not attempt to determine the exact grid position of the mobile user but the cell that the user is in.

### 3.2. Cell Training Data

After cell segmentation, a Wi-Fi database is created for each cell using the signal strength measurements collected during the training phase. Cell training data, which involves the RSS from all visible APs intensively sampled at multiple RPs within each cell and each RP is associated with manually labelled cell ID. The RPs are randomly selected within each cell and their locations do not need to be known.

Given a building with a set of cells *R*, the total number of visible Wi-Fi APs is *N*. For a given cell r∈R, a Wi-Fi measurement is a vector containing signal strength from *N* APs, denoted as:

(1) $$S_{\left(r,j\right)}=\left\{AP_{1}:Rss_{1,j},AP_{2}:Rss_{2,j},\cdots ,AP_{N}:Rss_{N,j}\right\},j=1,\cdots ,M$$

*M* is the total number of measurements at cell *r* and could vary by room. Each AP is identified by its unique MAC address and $Rss_{i,j}$ is the signal strength value from $AP_{i}$ in the $j_{th}$ measurement. Note the RSS value is replaced with *NaN* for the AP unobserved in one measurement.

During the offline phase, the signal strength from all visible APs are intensively sampled at multiple RPs within each cell. The training data for cell *r* fused from all RPs are stored in a M×N matrix denoted by $S_{r}=\left\{S_{\left(r,j\right)}|j=1,\cdots ,M\right\}$.

#### 3.2.1. Calibration

In this paper, we fuse crowdsourcing training data collected by multiple devices, which is the most promising solution for reducing the site survey labour consumption [43,44]. Most existing localisation systems assume that the device contributed for the training data collection is the same in positioning phase. However, every mobile user may become a potential contributor for the fingerprint database construction and the participating devices are usually different, which causes new challenges pertaining to cross-device fingerprint database construction. In addition, different devices have different sensor specifications and varying readings even at the same locations [45], thus a calibration process is essential in the crowdsourced radio map construction by fusing the RSS radio maps from different devices.

To support different devices and make the fingerprints of diverse devices compatible with each other, the calibration step is performed prior to the positioning phase. The relation in RSS values between two different devices at the same location appears to be linear, as discussed in [46]. In this case, device calibration is conducted by means of data fitting methods that create a linear transformation from the new device to the reference device. The adjusted RSS are then fused as crowdsourced training data.

In this paper, we implement the multivariate linear regression model [47] to match the signal strengths measured by the new device with the radio map constructed by the reference device. Calibration data collection is simple: the user carries the reference device and the devices that need to be calibrated; walks freely inside the area of interest; and collects data from all visible APs in the environment at the same time. Given a linear mapping with parameters $a_{mvregress}$ and $b_{mvregress}$, the signal strength values reported by Client X are mapped to the RSS values reported by Client Y. The linear regression model is expressed as:

(2) $$DeviceY_{RSSintensity}=a_{mvregress}\times DeviceX_{RSSintensity}+b_{mvregress}$$

where $DeviceX_{RSSintensity}$ is the RSS readings from Device *X* that needs to be calibrated, while $DeviceY_{RSSintensity}$ is the RSS readings from the reference Device Y. $a_{mvregress},b_{mvregress}$ are the calibration coefficients calculated by the linear regression model.

**Figure 3:**
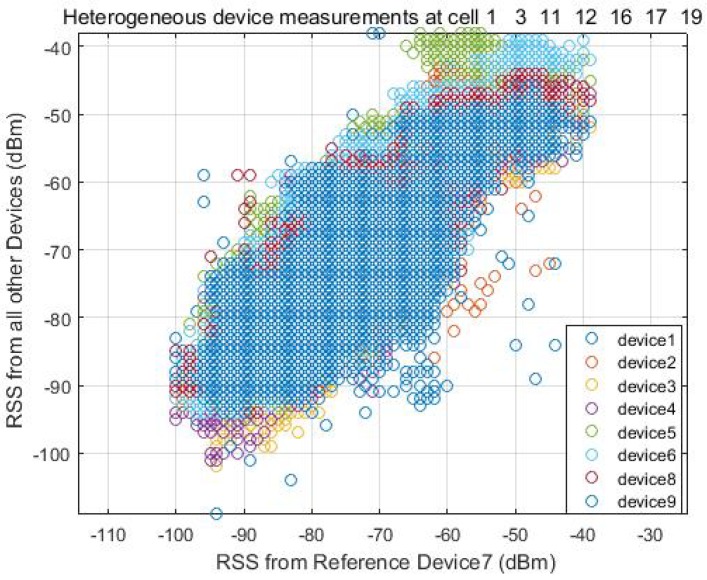
Heterogeneous devices measurements.

**Figure 4:**
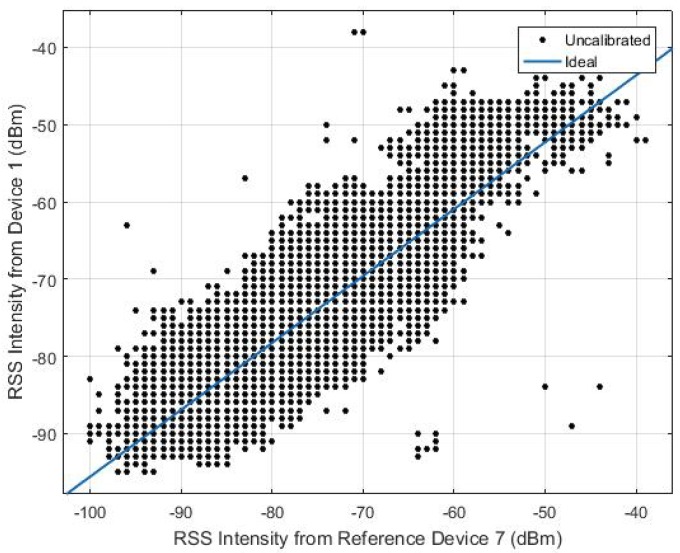
Multivariate linear regression between Device 1 and Device 7.

To find the linear fit between the two signal strength intensity, the user carries all devices freely walking around the test area and collects the RSS at the same time. In Figure 3, we can see the signal strength from all other devices almost follows a linear match with the reference signal strength intensity at every location. Figure 4 shows a calibration example between Device 1 and Device 7, where Device 7 is used as the reference device. The device specification is described in Table 1 in the Experimental Section 4.

#### 3.2.2. Missing Data Imputation

In a real open space environment, many APs can be scanned, which leads to a high-dimensional fingerprint database. Due to the RSS variability, APs may not be visible in every scan, leading to missing data. Missing data can reduce the statistical power of an investigation and produce biased estimates, leading to invalid conclusions [48]. Before we apply the MVGMM to estimate probabilistic fingerprint distributions for each cell, we need to handle the missing data problem.

In the case of the missing RSS values from some APs in RSS measurement vectors, the simplest way for imputing missing values is to set a constant, the lowest possible reading or the mean RSS value of each AP [49]. Howeverm this will alter the shape of the distribution and bias the covariance. A scenario may arise when RSS values from certain APs are available in the survey phase but are not observed in the online stage. A common approach is to find the effective APs which are visible in both the training and positioning phase [50]. The system in [51] created RSS reference surfaces for each AP using the Support Vector Regression (SVR) Machine to infer the missing data. Then, during the localisation stage, the measured RSS from each AP will be searched in the corresponding surface. In [52], a multilayer perceptron (MLP) artificial neural network (ANN) with fingerprinting approach has been investigated to handle the problem of missing APs in online matching stage. All the aforementioned approaches neglect the spatial correlation to simplify generation of theoretical RSS datasets for each missing APs in the offline phase, which will result in poorer localisation performance.

In this paper, we implement the expectation maximisation (EM) algorithm for incomplete data parameter estimation, assuming the missing data mechanism under the missing at random (MAR) assumption. A detailed description of the algorithm can be found in [53,54,55].

The EM algorithm is an iterative process that finds the maximum likelihood estimation (MLE) of the parameters until they converge in the presence of missing data. In general, the E (expectation) step calculates the expectation of the log-likelihood function given the observed data. The M (maximisation) step is to update the new parameters that maximise the expected log-likelihood from the E step. Suppose the complete dataset *Y* is partitioned into $Y=(Y_{obs},Y_{miss})$, where $Y_{obs}$ represents the observed part of *Y*, while $Y_{miss}$ is the missing part. The unknown parameter model θ of *Y* can be written as:

(3) $$P\left(Y|\theta \right)=P\left(Y_{obs},Y_{miss}|\theta \right)=P\left(Y_{obs}|\theta \right)P\left(Y_{miss}|Y_{obs},\theta \right)$$

Given an initial guess of $\theta^{\left(t\right)}$, it is possible to calculate the distribution of the missing data $P(Y_{miss}|Y_{obs},|\theta^{\left(t\right)})$. The E step is to calculate the expected complete data log-likelihood ratio $Q(\theta |\theta^{\left(t\right)})$ with respect to the imputation model of missing data.

(4) $$Q\left(\theta |\theta^{\left(t\right)}\right)=\int \log\left[p\left(Y_{obs},Y_{miss}|\theta \right)\right]P\left(Y_{miss}|Y_{obs},\theta^{\left(t\right)}\right)dY_{miss}$$

The M step maximises $Q(\theta |\theta^{t)})$ from the previous E step:

(5) $$Q\left(\theta^{\left(t+1\right)}\right)=\mathrm{arg max}\;Q\left(\theta |\theta^{\left(t\right)}\right)$$

### 3.3. Probabilistic Fingerprint

During the offline phase, a probability density function for each cell is estimated based on the MVGMM. Most current work that exploits GMM to estimate the probability distribution tends to specify a fixed mixture component, while it is important to note that the mixture component is a variable that is acting together to determine the overall estimation. The Akaike’s information criterion (AIC) measures the goodness of fit of statistical models [56] and it is applied in this paper to find the optimal number of components *K* of MVGMM. The authors decided that seven mixture components should be used in terms of optimum classification results and computation burden, as presented in the Optimal *K* Section 4.6. Then, an online matching process fits the online observation with the optimal parameters calculated during the offline process to identify the probability the observation belongs to each cell.

#### 3.3.1. Multivariate Gaussian Mixture Model

The probabilistic fingerprint is the conditional probability distribution of signal strengths given the cell position $P(S_{r}|r),r\in R$. The assumption of Gaussian distribution of the RSS is not accurate enough, as proven by Kaemarungsi and Krishnamurthy [57]. A Gaussian Mixture Model allows approximation of a probability density function by a weighted sum of Gaussian densities each with different parameters.

Most research work uses the GMM to approximate the RSS distribution for a single AP and ignores the interference between signals from different APs [58,59,60]. The MVGMM is implemented to approximate the probability density distribution of the training data for each cell, which takes advantages of correlation between the RSS from various APs within a certain area.

Given the training data *S* at cell *r* contains *M* RSS measurements from *N* APs, (for convenience, we remove the notation *r* in $S_{r}$ in the subsequent sections), and considering one measurement contains signals coming from *N* APs, the training data *S* are a matrix consisting of multivariate random variables. The density function modelled by MVGMM can be mathematically defined as:

(6) $$P\left(S|\mu ,\Sigma ,\pi ,r\right)=\sum_{k=1}^{K}\pi_{k}N\left(S|\mu_{k},\Sigma_{k}\right)$$

where *K* is the number of component of the model, $\sum_{k=1}^{K}\pi_{k}=1$. $\pi_{k},\mu_{k},\Sigma_{k}$ are the mixture weight, mean and covariance matrices for the $k_{th}$ mixture component. $N(S|\mu_{k},\Sigma_{k})$ is the $k_{th}$ mixture component from *N*-dimensional multivariate Gaussian distribution:

(7) $$N\left(S|\mu_{k},\Sigma_{k}\right)=\frac{1}{{2\pi }^{N/2}{\left|\Sigma_{k}\right|}^{1/2}}\exp^{-\frac{1}{2}{\left(S-\mu_{k}\right)}^{T}\Sigma_{k}^{-1}\left(S-\mu_{k}\right)}$$

During fingerprinting, the signature of cell *r* is generated by the MVGMM parameterised by $\Phi =\{\mu_{k},\Sigma_{k},\pi_{k}\},k=1,\cdots ,K$. The EM algorithm is applied to estimate the parameters of the model.

(1) E step. Calculate the responsibilities using the current parameters, which can be viewed as the posterior probability that the $m_{th}$ measurement $S_{m}$ is from the $k_{th}$ component.
  (8) $$\gamma_{k}\left(S_{m}\right)=\frac{\pi_{k}N\left(S_{m}|\mu_{k},\Sigma_{k}\right)}{\sum_{j=1}^{K}\pi_{j}N\left(S_{m}|\mu_{j},\Sigma_{j}\right)}$$
(2) M step. Re-estimate the parameters using the responsibilities from the E step.
  (9) $$\mu_{k}^{*}=\frac{1}{M_{k}}\sum_{m=1}^{M}\gamma_{k}\left(S_{m}\right)S_{m}$$
  (10) $$\Sigma_{k}^{*}=\frac{1}{M_{k}}\sum_{m=1}^{M}\gamma_{k}\left(S_{m}\right)\left(S_{m}-\mu_{k}^{*}\right){\left(S_{m}-\mu_{k}^{*}\right)}^{T}$$
  (11) $$\pi_{k}^{*}=\frac{M_{k}}{M}$$
  where
  (12) $$M_{k}=\sum_{m=1}^{M}\gamma_{k}\left(S_{m}\right)$$
(3) Evaluation. Evaluate the log-likelihood
  (13) $$lnP\left(S|\mu ,\Sigma ,\pi \right)=\sum_{m=1}^{M}ln\left\{\sum_{k=1}^{K}\pi_{k}N\left(S_{m}|\mu_{k},\Sigma_{k}\right)\right\}$$
  These three steps are iteratively repeated until the log likelihood convergences.

#### 3.3.2. Conditional Probabilistic Observation Model

Most work ignores the unobserved RSS value in the runtime observation, while we investigated that the missing APs also provide extra beneficial information in deciding the user position because of their “*unseen*” properties. This paper presents a conditional probabilistic likelihood observation function, by taking advantages of the invisibility of APs, referring to the likelihood of observing *a particular invisible AP set*.

The hypothesis is that, if an AP cannot be scanned for the whole training data collection within cell *r*, then an online observation contains RSS value from that AP would have low probability belongs to that cell. In other words, if an observation contains RSS values from the APs that *should not to be seen in cell r*, then the probability of being located in cell *r* given the observation would be lower. This is expected as the APs with no RSS readings are less probable to be heard within the same area.

By splitting the observation *O* into $O_{RSS}$, the RSS measurements for the visible APs, and $O_{Z}\left(I\right)$, a binary indicator variable for APs, $O_{Z}\left(i\right)=1$ if AP *i* is invisible and 0 otherwise. In this case, we define the *particular invisible AP set I* as:

(14) I=∩(Invisible APs r, Visible APs O)

The observation probability would be:

(15) $$\begin{array}{ccc} P(O|r) & = & P(O_{RSS},O_{Z}\left(I\right)|r) \end{array}$$

(16) $$\begin{array}{cc} = & P\left(O_{RSS}|O_{Z}\left(I\right),r\right)P\left(O_{Z}\left(I\right)|r\right) \end{array}$$

$P(O_{RSS}|O_{Z}\left(I\right),r)$ matches the online observation with the probabilistic fingerprint discussed in Section 3.3.1. $P(O_{Z}\left(I\right)|r)$ is a likelihood of observing RSS from the invisible AP sets of cell *r*.

(17) $$P\left(O_{Z}\left(I\right)|r\right)=\prod_{i=1}^{P}P\left(O_{Z}\left(i\right)|r\right)$$

(18) $$P\left(O_{Z}\left(i\right)|r\right)=\frac{O_{Z}^{r}\left(i\right)}{\sum_{r\in R}O_{Z}^{r}\left(i\right)}$$

where P=|I|. $O_{Z}^{r}\left(i\right)$ is the invisibility of AP *i* at cell *r*, and $\sum_{r\in R}O_{Z}^{r}\left(i\right)$ is the invisibility of AP *i* over all cells *R*.

### 3.4. Hidden Markov Model

The motion of the user can be modelled as a Markov process [61] and a HMM is applied to track the mobile user, where the hidden states comprise the possible cell locations and the RSS measurements are taken as observations.

**Figure 5:**
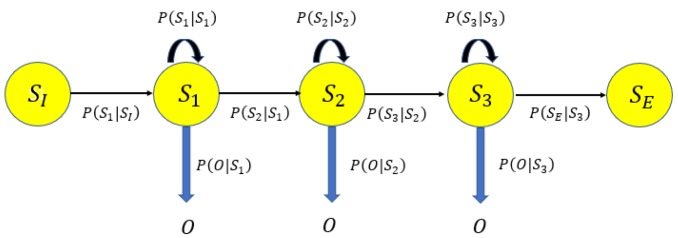
HMM.

The formal definition of a HMM is as follows, depicted in Figure 5. The set of states are identical to the set of cells. Let $S_{1},S_{2},\cdots ,S_{T}$ be the sequence of hidden states in the state set *R* during a time sequence t=1,⋯,T, which constitutes the user moving trajectory. The observed Wi-Fi RSS sequence $O=O_{1},O_{2},\cdots ,O_{T}$ up to time T in correspond. The model is characterised by parameters λ={A,B,α}.

Given an observation sequence, the Viterbi algorithm determines the most probable hidden state sequence.

(19) $$P\left(O|\lambda \right)≃\max_{S_{1},S_{2},\cdots ,S_{T}}P\left(O_{1},O_{2},\cdots ,O_{T},S_{1},S_{2},\cdots ,S_{T}|\lambda \right)$$

A is the transition probability matrix. The segmentation rule based on the building topology is encoded in the state transition probability, which is the probability of the user moving from *cell i* to *cell j*, denoted as $p_{i,j}=P\left(S_{t+1}=S_{j}|S_{t}=S_{i}\right)$. If a given cell is linked to *n* other cells (including itself), then the probability of moving to one of these cells is defined to be 1/n, and the probability of moving to other isolated cells is 0. Here, we use equal probability for simplicity.

B is the emission probability, i.e., the likelihood of producing observation $O_{t}$ from *cell $S_{j}$*, which is to fit the observation to the signature of each cell calculated by the MVGMM and the conditional observation likelihood, referring to Equations (6), (15) and (17):

(20) $$b_{j}\left(O_{t}\right)=P\left(O_{t}|S_{t}=S_{j}\right)=\sum_{k=1}^{K}\pi_{k,j}N\left(O_{t}|\mu_{k,j},\Sigma_{k,j}\right)\times \prod_{i=1}^{P}P\left(O_{Z}\left(i\right)|S_{j}\right)$$

where $\Phi_{j}=\left\{\mu_{k,j},\Sigma_{k,j},\pi_{k,j}\right\},k=1,\cdots ,K$ is the mixture parameters associated with *cell $S_{j}$*. α is the prior state probability; here, we assign equal prior probability to each state.

## 4. Experimental Results

To verify the proposed approach, a field test was carried out on Level 2 of the Bolz Hall, Civil & Environmental Engineering building, Ohio State University, United States. The geometry of the building consists of labs, offices and classrooms, as shown in Figure 2. We divided the floor plan into 20 cells on topology. Typically, there is one cell per room. We also segmented the two long hallways into cells, which are Cells 1–4 and Cells 5–7 denoted in Figure 2. The first hallway connects the entrance of the building to the test area and the second corridor connects the right hand side eight administration offices (Cells 13–20) to the main hallway. All training data collection took place during five days covering different times of the day. During the collection, students and staffs walked around normally as usual.

We analysed the correlation between the RSS measurements in the training data for each cell, and presented the efficiency of the proposed localisation system for both stop and go movement and dynamic walking data. The minimum training size and the affect of different *K* mixture component were investigated to attain certain room level localisation accuracy. A comparison was carried out between the mean RSS and the EM imputation method in terms of replacing the missing values in the training data.

In the filed test, nine android devices were used for the crowdsourcing data collection (see Table 1). For Wi-Fi data collection, the CPS App developed by Mr. Hofer was used [62]; each Wi-Fi scan records the timestamp, location ID, MAC address, network name and RSS values for all visible APs. The devices collected signals from the university public base stations about which we had no prior information. The data collection consisted of three stages: calibration, static training and real kinematic walking data collection.

Calibration data collection was conducted to get the coefficients for each device with respect to the reference device (see Section 3.2.1). The nine devices were put on a trolley and one user pushed the trolley around the test area and stopped at random in various cells. Each device collected 200 scans at each location. In this paper, we use Device 7 as the reference device, and the calibration coefficients for the other seven devices are displayed in Table 2. The calibrated RSS measurements for each device is calculated as:

(21) $$\tilde{RSS_{d}}=M_{d}\times RSS_{d}+C_{d}$$

where $RSS_{d}$ is the raw RSS measurements taken by device *d*, $M_{d},C_{d}$ are the calibration coefficients and $\tilde{RSS_{d}}$ is the calibrated RSS measurements for device *d*.

After segmentation, each device was randomly assigned multiple RPs (normally 5–10) within each cell to collect training data in static mode. Note that the locations of the RPs were physically different against each device. Then, the training data for each cell is obtained by fusing the data collected at all the RPs from every available device. The locations of the RPs need not to be known, and every device generally chose different times to enter into the cell to assure that the training samples are covering the whole space and time variant features. At each RP, each device was designed to collect 200–400 scans in static mode; each scan records the timestamps, point ID, MAC address, network name and RSS values for all visible APs in the environment.

### 4.1. AP Interdependency Analysis

To verify the interdependence of RSS from various APs, we compared the static data collected at a single point by one device with the training data of the corresponding cell collected by the same device at multiple RPs. Intuitively, we chose the three strongest APs (APs 19, 57, and 74) for the two datasets to do the analysis.

From the results shown in Table 3, the RSS properties of the three APs are quite distinct at a single point from within a room. Regarding the point data, the RSS values are more stable and have smaller variations compared with the cell data. The correlations between pairs of APs at RP1 are as small as 0.086 which is similar to the results given in [57], while the correlation can also be as large as −0.27 in complex, noisy and non-line-of-sight signals. In the cell data, the correlations between pairs of APs become so large that we can no longer assume the RSS samples from the visible APs are independent, which also explains why the proposed algorithm consider the correlations from RSS measurements between pairs of APs.

### 4.2. AP Density

**Figure 6:**
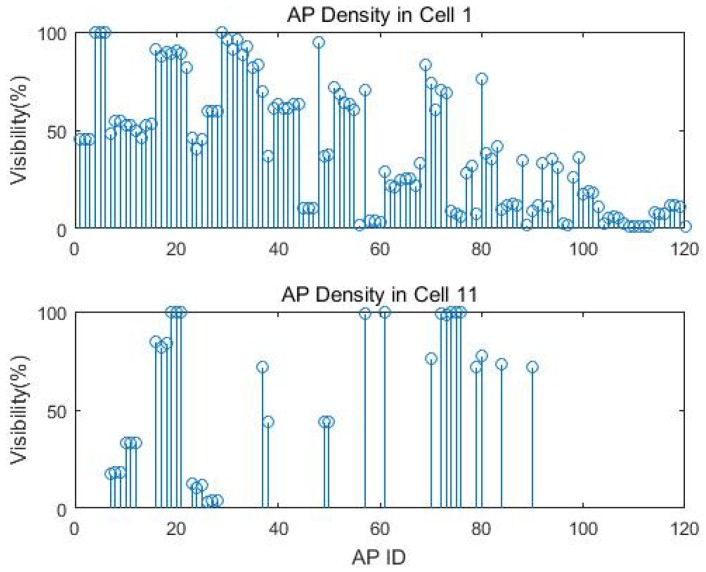
AP density and missing data percentage at Cells 1 and 11.

The exact number of training samples for each cell is displayed in Table 4. Cell 1 had the most visible APs with 120, while Cell 11 had the fewest visible APs with 34. The visibility of each AP means the number of observation from the AP compared to the total number of measurements. These 120 visible APs were *registered* in the training data and used to extract the invisible AP sets for each cell. Figure 6 gives the example of the AP intensity and the missing data percentage for Cells 1 and 11. We can clearly see that different cells have distinct visible and invisible AP sets. The missing data percentage can be as high as 98.87% for Cell 1 and 99.92% for Cell 11 from AP 109 and AP 78, respectively. We manually removed APs with less than 1% visibility before applying the EM imputation to avoid singular covariance.

### 4.3. Stop and Go Localisation Accuracy

In this section, we present the analytic results that were calculated using the observations obtained during the training phase as inputs to the location system. We chose 500 scans randomly out of the crowdscourced training samples for each cell and excluded them from the training data. The remaining set was used to train the MVGMM model and get the probabilistic fingerprint for each cell. The 500 scans we removed from the training data were formed as the test set for each cell. Note the 500 test scans could be from any test device.

We constructed the stop and go movements by including transition between cells; the observed RSS sequences were simulated by randomly choosing 50 scans from the 500 test samples of each cell.

In the stop and go tests, nine different trajectories were designed to verify the proposed algorithm. The first six trajectories were designed to move only between adjacent cells, covering different parts of the test area. While Trajectory 7 was designed to repeat Trajectory 4 but miss transition data at three cells. Trajectory 8 was designed to repeat Trajectory 5 but miss more transition data at six cells. Trajectory 9 repeated Trajectory 6 with data missing for eight cell transitions. These latter three trajectories were selected to simulate the scenarios that continuous RSS measurements cannot be obtained for a period of time during the transition between cells. This is reasonable since one Wi-Fi scan can take around 3 s for some devices, while the user has already passed the transition cell.

In the following analysis, the number of component *K* was set to K=7. Table 5 gives the average matching accuracy for the designed nine trajectories. We performed the experiments 50 times for each trajectory, randomly choosing different test samples each time. The proposed system can still work properly when the system failed to get updated observation data for a certain time, referred to the tracking results of Trajectories 7–9. The accuracy decreased if the observation data in the transition cell is missing, however, the HMM based algorithm can still recover from the losing track of position with an average matching accuracy of 97.11%. Matching accuracy is defined as the percentage of the cells correctly determined:

(22) $$Accuracy=\frac{\sum_{t=1}^{T}Equal\left(s_{t}^{HMM},s_{t}^{True}\right)}{\left|T\right|}$$

where

$$Equal\left(a,b\right)=\left\{\begin{array}{cc} 1 & a=b\\ 0 & otherwise \end{array}\right.$$

**Figure 7:**
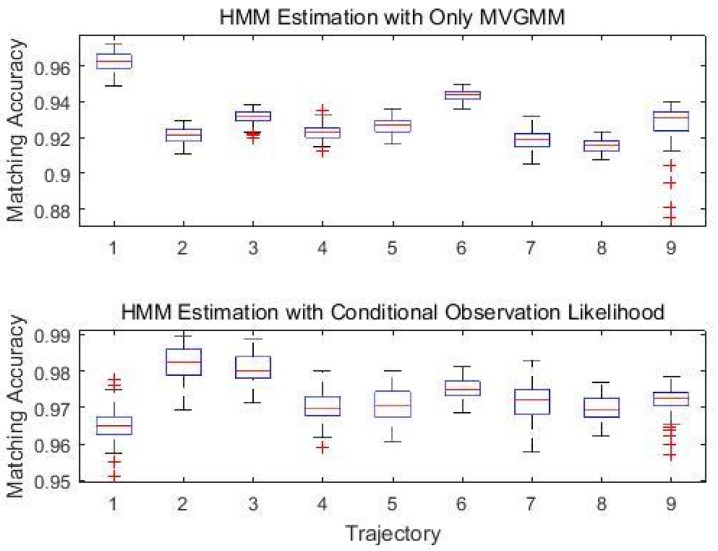
Conditional likelihood function contributes to the improvement of localisation performance.

With the aid of the proposed conditional likelihood observation function that utilises the information of the invisible APs, the system can achieve an average of 97.29% matching accuracy even when the observed data are not continuous. Figure 7 demonstrated efficiency of the distinct invisible AP set of different cells being a significant signature which helps to improves the localisation performance from an average 92.98% matching accuracy to an overall 97.29% matching accuracy.

### 4.4. Kinematic Tracking Accuracy

In addition to the stop and go simulated movement, we also conducted dynamic experiments on some devices to track a moving agent that freely moves around with normal walking speed. The user was asked to press the “*checkpoint*” button in the CPS App to record the timestamps every time he entered into a new cell. Both the checkpoint time and Wi-Fi scan time use the same nano time of the android system.

**Figure 8:**
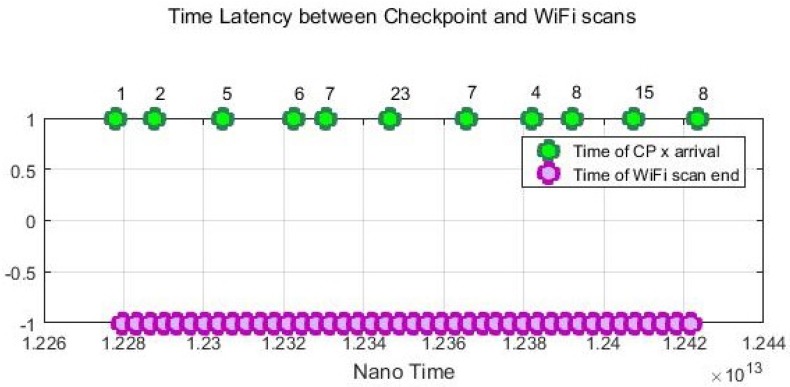
Time latency between checkpoints and Wi-Fi scans.

Figure 8 gives an example of the recorded Wi-Fi scan time sequences of Device 4. The average time difference between the checkpoint time and the Wi-Fi scans is 1.22 s. In Figure 8, there are some latencies between the checkpoint timestamps and the Wi-Fi scans since one Wi-Fi scan can take 0.6–3.7 s depending on devices. Considering that a Wi-Fi scan can take few seconds and a user can change the position while scanning is done. Thus, while a user is moving across cells, there will always be a blur in the Wi-Fi scan and the exact cell ID.

**Figure 9:**
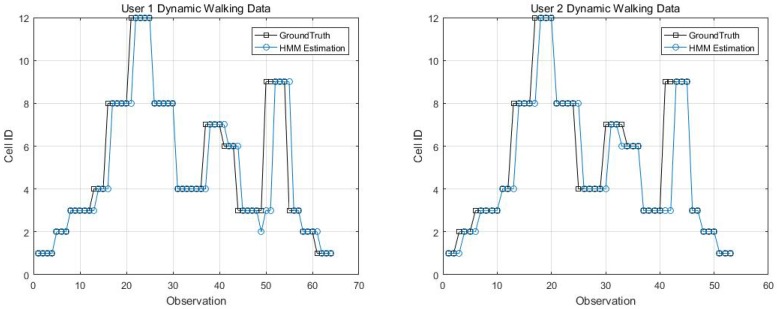
Kinematic Trajectory 1 Repeated by Device 1 and 2.

**Figure 10:**
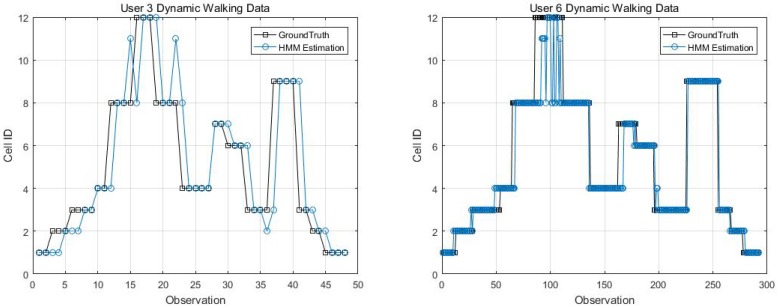
Kinematic Trajectory 1 repeated by Device 3 and 6.

**Figure 11:**
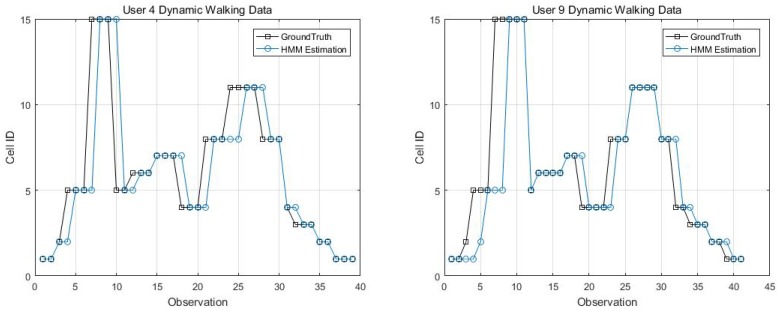
Kinematic Trajectory 2 repeated by Device 4 and 9.

Two kinematic trajectories were designed and repeated by different users. Each trajectory was defined as the sequence of the cell IDs along with the movement. Devices 1, 2, 3, 6 and 7 repeated Trajectory 1, while Devices 4 and 9 repeated Trajectory 2. Seven users carried the devices starting from the same cell (normally started from Cell 1), and repeated each trajectory several times. Note that the real walking trajectory can be different, as the user can walk into different locations within each cell. Here, we only show part of the results due to the limited space, see Figure 9, Figure 10 and Figure 11.

### 4.5. Training Size

Collecting enough data for creating location statistical fingerprints is the key to achieving good performance. As pointed out by Zhou [63], for a grid localisation system, 5–6 APs deployed strategically within the test area would be ideal and each location should have enough calibration samples (e.g., 200–300 samples). To evaluate the performance of the proposed system with smaller training samples, we chose different training sizes ranging from 10% to 100% of the collected training data.

**Figure 12:**
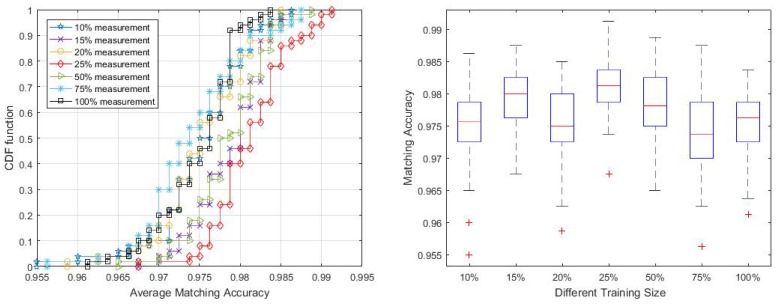
Matching accuracy in dependence of training size.

The plot in Figure 12 shows that, with 25% of measurements, the method can achieve the best performance with 98% accuracy in over 50% of the trials. Generally, all sample sizes are enough to train the MVGMM and can get over 97% matching accuracy for half of the trials. However, we also noticed in Figure 12 that the proposed algorithm is insensitive to the size of the training samples, even presenting more robust localisation accuracy to lower sample sizes. This result is similar to the analysis in the work of Zhou [63] who found that, given denser calibration samples for the area may introduce more noise to distinguish from other areas. Elnahrawy et al. [64] also pointed out that, given larger training samples, it is unlikely that additional sampling will increase accuracy. The possible reason might be that larger training data contain more time-varying features and signal interference from the environment. We observe that approximately 15%–25% of training data per cell is sufficient to attain comparable level of accuracy.

### 4.6. Optimal *K*

**Figure 13:**
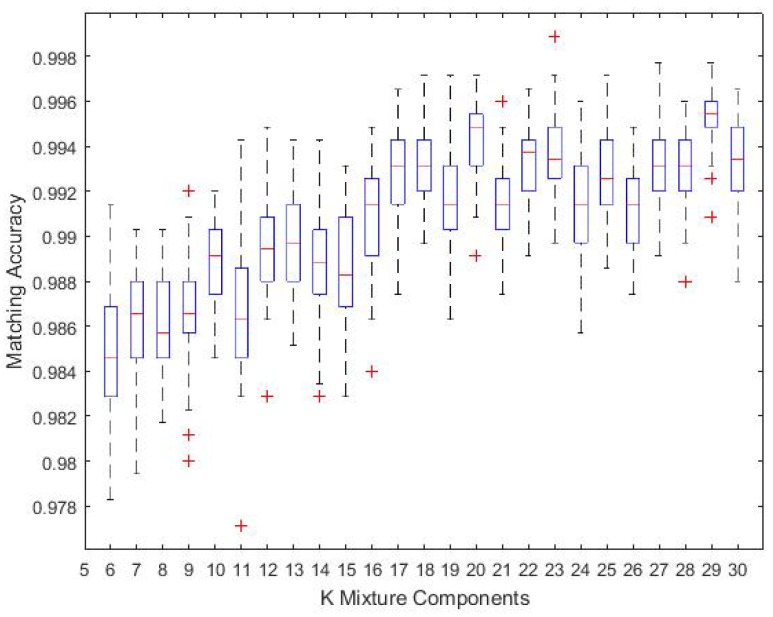
Optimal K in dependence of matching accuracy for Trajectory 6.

To understand the optimal *K*, we have pre-defined different thresholds when applying the AIC rule. Figure 13 presents a plot of the average matching accuracy of Trajectory 6 with *K* values of 6–30 based on 25% training data; each *K* value was run 50 times. Choosing a larger *K* will increase the accuracy to some extent with the cost of adding computation complexity. Thus, we set K=7 for computation simplicity purposes while maintaining reasonable localisation accuracy.

### 4.7. Comparison with Mean RSS Imputation

The proposed system applies the EM imputation method to deal with the missing data in the training set. This section explores the performances of the two missing data imputation methods. The only difference is the missing data in the training samples are replaced with the average RSS value for the corresponding AP.

Table 6 gives good averaged results even using the mean RSS imputation for the stop and go movements, although the accuracy is always worse than the one with the EM imputation.

**Figure 14:**
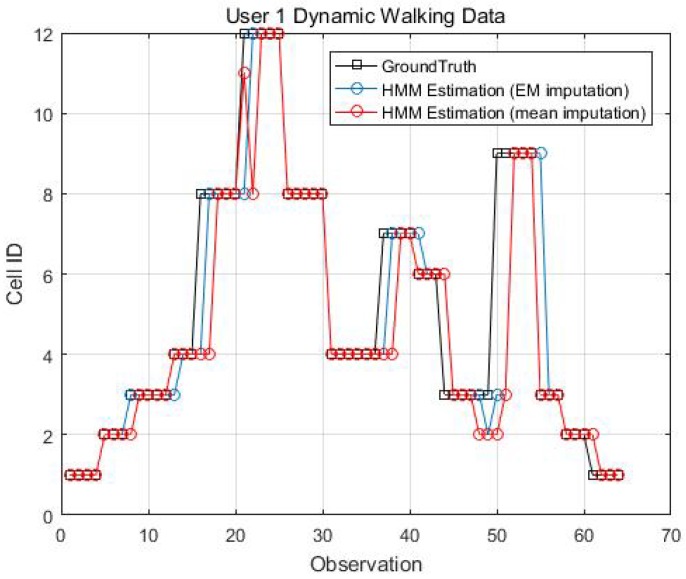
EM imputation vs. mean RSS imputation: User 1 dynamic walking data.

**Figure 15:**
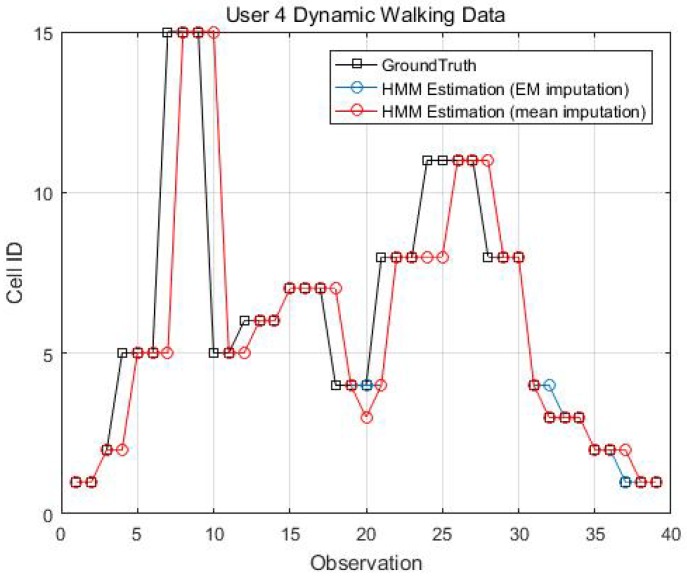
EM imputation vs. mean RSS imputation: User 4 dynamic walking data.

**Figure 16:**
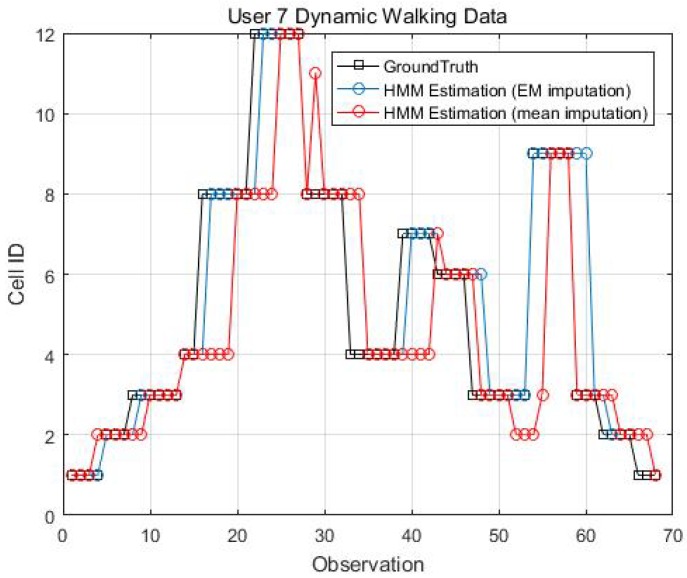
EM imputation vs. mean RSS imputation: User 7 dynamic walking data.

**Figure 17:**
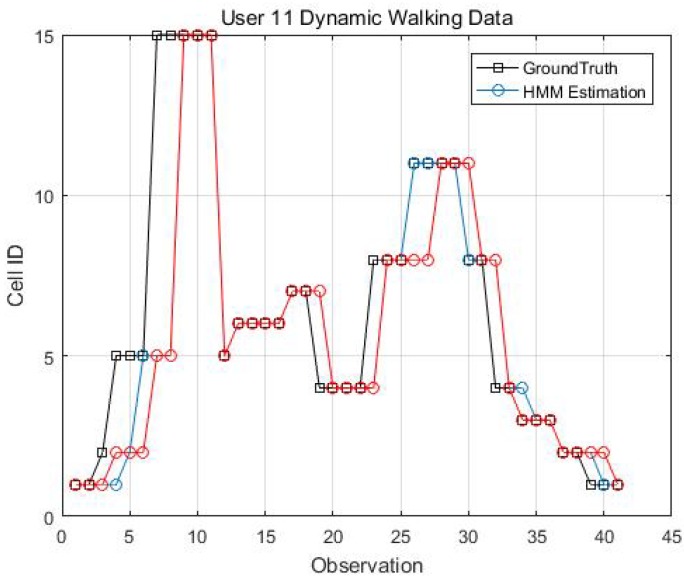
EM imputation vs. mean RSS imputation: User 9 dynamic walking data.

Figure 14, Figure 15, Figure 16 and Figure 17 display the comparison results of the kinematic walking data. The mean imputation can still maintain good accuracy, as the estimated trajectory almost matches with the ground truth with some latencies, although they normally have larger bias estimation than the EM imputation ones. In addition, the figures below clearly demonstrate the efficiency of the proposed conditional likelihood function which can help to distinguish the adjacent cells and correct the position.

## 5. Discussion

In this paper, we have validated the efficiency of the proposed conditional likelihood observation function. It correctly identifies the user’s position in most cases. However, in some cases, when the set of invisible APs for one cell is a subset of the invisible APs for another cell, no performance increase is observed.

There are some approaches dealing with the problem of the GMM parameter estimation based on incomplete data directly instead of replacing the missing ones before training [65]. The EM imputation is based on the assumption that the distribution is multivariate Gaussian, which still gives reasonable results, as presented in the paper. The implementation of such algorithm would be one of the future interests to quantify the improvement in the context of the current work.

Localisation accuracy in dependence of increasing training sample size is commonly discussed in the literature. We have a different observation based on the campus wireless data verification results. The possible reason may be the crowdsourcing training data contains large variations and interference from other signal channels in the campus wireless network. To avoid over-training, the Gamma Test [66] will be applied to identify the optimal training data size preventing performance from degenerating.

Cell 1 is a transition cell between the indoor and outdoor environment, which shows special characteristics in correspondence. At Cell 1, the system can see 120 maximum visible APs. The property that many APs are only visible at Cell 1 but invisible at all the other cells can be used to analyse the transition data between indoor and outdoor.

## 6. Conclusions

In this paper, we propose a statistical approach to localise the mobile user to room level accuracy based on university wireless network. The users have no basic knowledge about the base stations deployed within the environment in advance. The MVGMM is efficient at approximating the RSS distribution for each cell that takes the signals correlations into computation. The system obtained a reliable 92.98% matching accuracy for half of the trials based on the crowdsourcing data.

The performance can be improved to 97.29% by introducing the conditional likelihood observation function, which takes advantages of the *unseen* signatures of APs. Instead of ignoring the invisible APs, which are unobserved in the training data or the new observation, this paper investigated a conditional likelihood observation model calculated at each cell for all APs inclusive of the invisible ones, referring to a likelihood of observing an AP that is not supposed to be visible.

The proposed system demonstrates a practical prototype model of a reliable room location awareness system in a real public environment. It can handle the data uploaded by diverse devices and the noisy environment which can be widely applied in potential public spots such as guiding customers in a shopping mall or monitoring patients in a hospital. The system can be applied to a wide range of localisation applications in a practical indoor environment regardless of the quality of the signals, the number of the APs, the heterogeneous devices, the interference from other channels, the time-varying phenomena or the complexity of the environment.

## Figures and Tables

**Table 1:** Device specification.

Device ID	Brand	Average Time for One Scan (s)
1	Samsung S8	2.87
2	Samsung Galaxy A6	3.565
3	Samsung S3	3.665
4	Samsung S3	3.46
5	Google Pixel	3.50
6	Moto G3	0.575
7	Huawei Mate 7	2.55
8	Oneplus 1	3.06
9	LG G4	3.355

**Table 2:** Device calibration coefficients.

Device ID	*M*	*C*
1	0.9336	−6.7754
2	0.8825	−11.4863
3	0.8951	−8.7945
4	0.8600	−11.9044
5	0.7802	−21.3151
6	0.8709	−13.8607
8	0.9706	−5.3788
9	0.8701	−10.6396

**Table 3:** Correlation between APs (single point vs. cell).

RSS Properties	RP1 (Device 6)	Cell 11 (Device 6)
Number of scan	400	4000
Number of visible APs	21	23
Mean RSS of AP19 (dBm)	−66.90	−65.42
Mean RSS of AP57 (dBm)	−78.94	−78.95
Mean RSS of AP74 (dBm)	−69.33	−65.08
standard deviation of AP19 (dBm)	1.82	3.29
standard deviation of AP57 (dBm)	0.84	3.05
standard deviation of AP74 (dBm)	1.87	3.27
Correlation (AP19, AP57)	0.086	0.21
Correlation (AP19, AP74)	−0.27	0.50
Correlation (AP57, AP74)	−0.18	0.37

**Table 4:** Training sample size and visible APs for each cell.

Cell IDs	Training Data		Cell IDs	Training Data	
	Sample Size	Visible AP Number		Sample Size	Visible AP Number
1	12,000	120	11	10,000	34
2	12,000	94	12	12,000	38
3	11,001	85	13	10,100	62
4	12,004	67	14	4000	58
5	8000	82	15	5473	42
6	11,800	61	16	5500	42
7	12,000	61	17	7368	45
8	12,103	54	18	4500	53
9	11,000	55	19	4499	49
10	12,000	56	20	4800	47

**Table 5:** Matching accuracy for stop and go trajectories.

Trajectory	Number of Covered Cells	Acc with Conditional Observation Likelihood
1	16	96.53%
2	17	98.16%
3	21	98.04%
4	23	97.02%
5	25	97.05%
6	35	97.50%
7	20	97.20%
8	19	96.99%
9	27	97.16%
Average Accuracy	23	97.29%

**Table 6:** Mean RSS imputation vs. EM imputation.

Trajectory	Mismatch with Mean RSS Imputation	Mismatch with EM Imputation
1	5.10%	2.51%
2	3.53%	1.84%
3	3.68%	1.96%
4	4.05%	2.98%
5	3.85%	2.95%
6	3.29%	2.50%
7	3.96%	2.80%
8	4.32%	3.01%
9	3.94%	2.84%
